# Office Three-Dimensional Printed Osteotomy Guide for Corrective Osteotomy in Fibrous Dysplasia

**DOI:** 10.7759/cureus.36384

**Published:** 2023-03-20

**Authors:** Muhammad Fawwaz Zamri, Bing Wui Ng, Kamal Jamil, Abdul Halim Abd Rashid, Ahmad Fazly Abd Rasid

**Affiliations:** 1 Department of Orthopaedics and Traumatology, Universiti Kebangsaan Malaysia Medical Centre, Kuala Lumpur, MYS; 2 Department of Orthopaedics and Traumatology, Hospital Pakar Kanak-Kanak UKM, Kuala Lumpur, MYS; 3 Department of Orthopaedics and Traumatology, Faculty of Medicine, Universiti Kebangsaan Malaysia Medical Centre, Kuala Lumpur, MYS

**Keywords:** computerized tomography, 3d surgical guide, virtual surgery, limb-length discrepancy, fibrous dysplasia

## Abstract

Fibrous dysplasia is a benign condition but can lead to severe long-bone deformities. Three-dimensional (3D) printing technology is a rapidly developing field that has now been popularized to aid surgeons in preoperative planning. We report a case of hip deformity in a 21-year-old woman who suffered from fibrous dysplasia and underwent a corrective osteotomy. We utilized open-source 3D computing software for preoperative planning before producing an osteotomy guide to aid in the operation.

## Introduction

Three-dimensional (3D) printing has been widely used in implant manufacturing and instrument innovation. However, this technology was limited by its high cost and complexity of processing. With current advancements, affordable open-source 3D design software and office 3D printers are widely available. Orthopedic surgeons could carry out preoperative planning for deformity corrective surgery in a 3D plane, with the added advantage of simulating the solutions and visualizing the anticipated outcome spontaneously.

Fibrous dysplasia is a bone disorder that happens when normal lamellar bones are replaced by cellular fibrous tissue with flecks of osteoid and woven bone [1]. Proximal femoral deformity or better known as ‘shepherd’s crook’ deformity is one of the commonest presentations of fibrous dysplasia [2]. Correction of proximal femoral deformity poses a challenging task. Common complications related to this procedure include bleeding, long operative time, and non-union.

We present a case of fibrous dysplasia with hip deformity and limb length discrepancies managed with corrective osteotomy aided by 3D design software in the preoperative stage and a patient-specific 3D-printed guide for osteotomy of the hip.

## Case presentation

**Figure 1:**
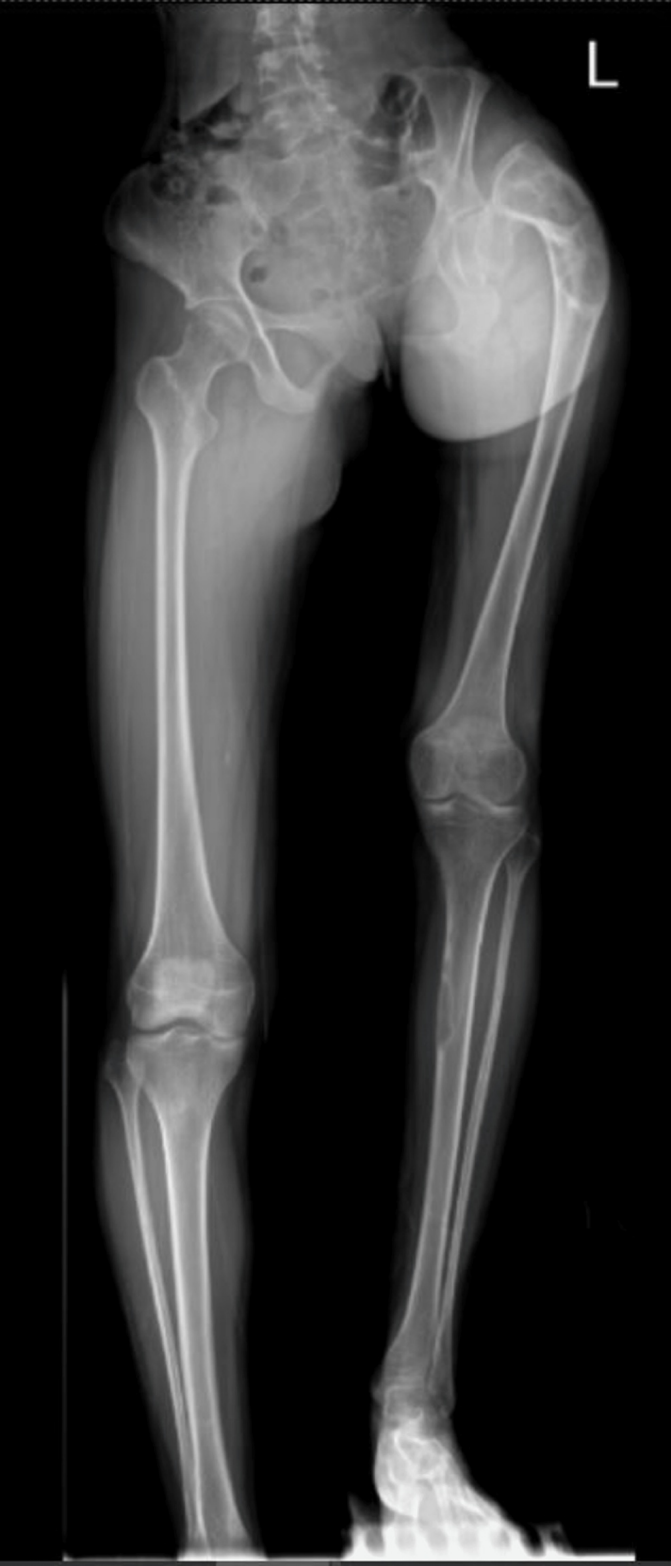
Preoperative plain radiograph of the lower limb showing severe limb length discrepancy caused by the shepherd's crook deformity of the left proximal femur.

A 21-year-old woman presented to our clinic with an abnormal gait and worsening left hip pain. She had had a background of polyostotic fibrous dysplasia since childhood. On physical examination, she walks with a short-limb gait. The Trendelenburg sign was positive on the left side. Her true length of the left lower limb is measured to be 11cm shorter than the right lower limb. The hip radiograph showed a neck shaft angle of 95° with lateral bowing of the left hip, typical of the 'shepherd's crook' deformity (Figure 1).

**Figure 2:**
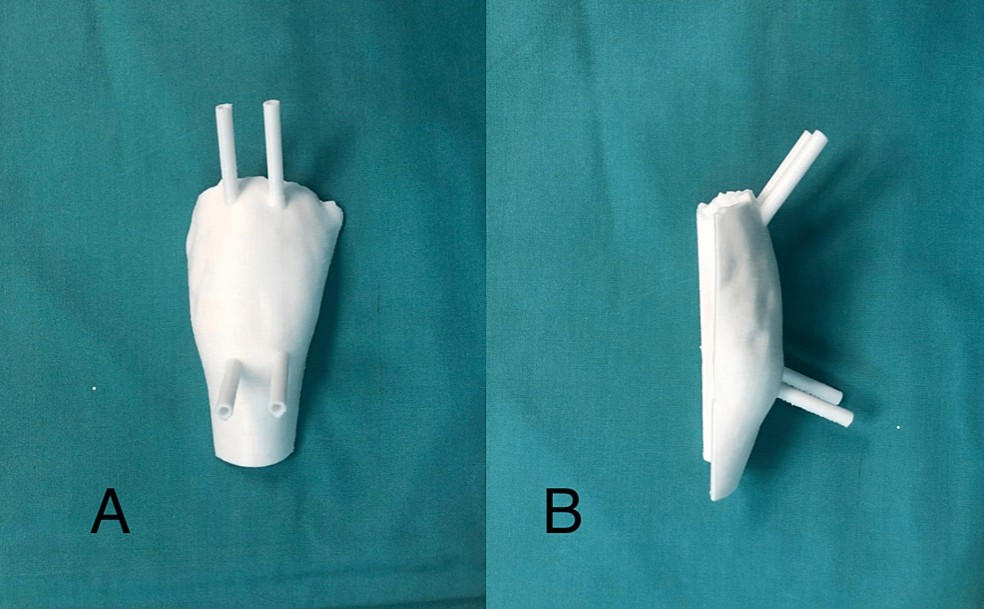
The front (A) and side (B) view of the 3D surgical guide printed using the PLA material. The surface of the guide is specific to the patient's proximal femoral anatomy. Four drill holes were designed to allow the placement of K-wires. 3D: Three-dimensional; PLA: polylactic acid; K-wires: Kirschner wires

A computerized tomography (CT) scan of her left femur was done and converted to DICOM images to enable preoperative planning with an open-source 3D software, Meshmixer (2020, Toronto, Canada). Virtual surgery was performed using the same 3D software. The angle needed for osteotomy was calculated to be 50 degrees after performing a virtual osteotomy on the femur. A patient-specific osteotomy guide was fashioned using the same software (Video 1). The guide design then underwent postprocessing using Slicer (2005, Massachusetts, USA). It was printed using an Ender Pro 3D printer with polylactic acid as the base material (Figure 2). Before surgery, the patient's femur and the guide were printed, the osteotomy was done, and the result was confirmed.

**Video 1:** Workflow of the surgical guide production based on patient-specific CT images. CT: Computerized tomography

**Figure 3:**
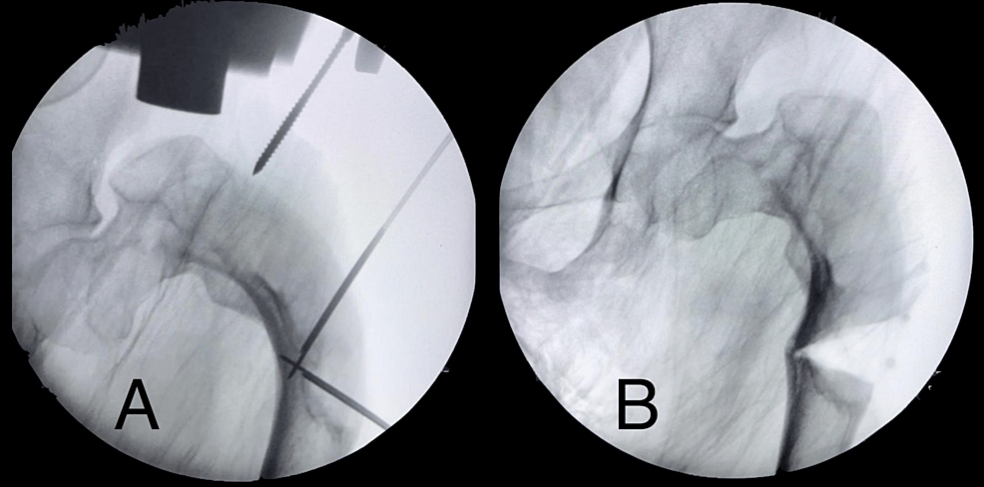
Intraoperative image intensifier images showing placement of K-wires before osteotomy (A) and after osteotomy (B). K-wires: Kirschner wires

**Figure 4:**
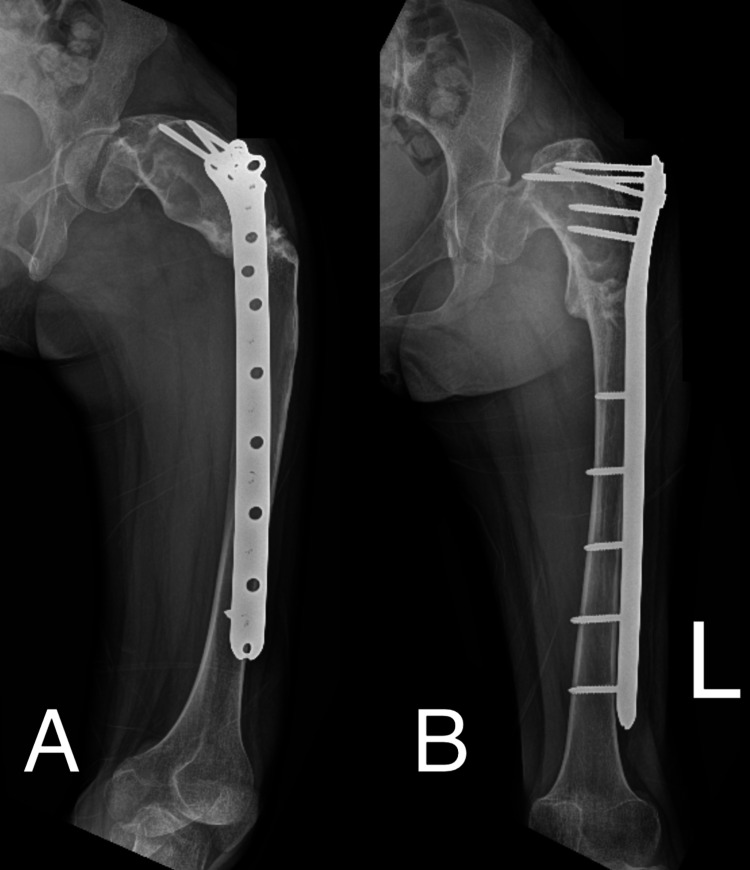
Radiograph showing lateral (A) and AP (B) view of the left lower limb during the first-year postop follow-up. AP: Anteroposterior

On the day of surgery, the guide was submerged in ortho-phthalaldehyde solution (Cidex®) for 15 minutes before being used on the patient. The patient was supine under general anesthesia, and the proximal femur was exposed through the standard lateral approach. The guide was placed over the deformity, and Kirschner wires (K-wires) were inserted through the prepared drill holes. The planned osteotomy site was checked using an image intensifier (Figure 3). The K-wires were then removed, leaving behind the drilled path. The guide was removed, and K-wires were reinserted following the prepared path to guide the osteotomy. The osteotomy was done using a combination of an oscillating saw and an osteotome. The osteotomy site was closed and fixed with a distal femur locking plate. No immediate postoperative complications were noted. The patient was discharged well with non-weight-bearing crutches for three months. Postoperatively, the limb length discrepancy has reduced to 4cm. This discrepancy was further overcome by using a shoe raise. Her hip pain reduces significantly post-op. The latest radiograph of her left femur shows a good union at the osteotomy site (Figure 4). The patient has been followed up for almost 18 months postop, and no further complications were noted.

## Discussion

3D technology has been widely used in tackling complex deformities recently. As the technology grows, 3D printing becomes more cost-effective and patient-specific, thus making it more sustainable in the future, especially in the areas of dentistry, traumatology, complex orthopedic oncology, and revision surgery [3-5]. Various authors have also produced case studies of patients with various deformities who benefited from this ‘do-it-yourself’ technology [6-8]. We believe that this case report is the first to describe the use of 3D software for virtual surgery and a 3D-printed surgical guide in managing shepherd’s crook deformity.

Effective preoperative planning is essential to ensuring a good outcome. The ability of 3D design software to process DICOM images obtained by CT enables clinicians to have a clearer understanding of the complex anatomy of a diseased region [9]. 3D visualization of anatomical features can be viewed from any angle, allowing a comprehensive assessment of the anatomy. Prior to this, two-dimensional (2D) radiograph templating was done for preoperative planning for corrective surgery of shepherd's crook deformity [10]. A previous study has also shown that 2D templating for arthroplasty lacks accuracy due to the difference in magnification of the radiograph [11].

In this case, 3D design software was used to simulate the desired correction and osteotomy angle. In the process of performing the virtual surgery, the osteotomy guide could be designed to fit exactly to the patient’s bone. Virtual surgery enables surgeons to simulate multiple solutions and visualize the impact of those solutions in real-time to avoid unnecessary risks to patients during surgery [12].

The ability to produce an exact-sized, patient-specific 3D-printed model of the bone and guide has brought forth further possibilities. In this case, the surgeon could simulate the surgery in real-time on the life-size model using the printed guide and femur model. The surgeon could have a feel for the workflow of the osteotomy and familiarize himself beforehand with the complexity of the real-size anatomical structure [13]. This, coupled with the patient-specific surgical guides, also enables the surgery to be done with high accuracy and, at the same time, reduces operative time, radiation exposure, the risk of bleeding and infection, and postoperative pain [14-16]. Reducing operative time also translates to reduced operating room costs [17]. Others have also used a similar technique to practice complex surgery and determine the exact implant size prior to surgery [13,18]. Besides that, the life-size models could be used for educational purposes in enhancing understanding for both patients and their families regarding the operative procedure prior to surgery.

In addition to the benefits mentioned in this case report, this technology has been increasingly used in instrument design and patient-specific 3D-printed implants [19]. However, there are concerns regarding the software quality used to process patient images and simulate virtual surgery. Further collaboration among industries is needed to ensure patient safety [20].

## Conclusions

3D printing is a reliable tool, especially in managing complex deformities. More comprehensive preoperative planning and simulation can be performed to reduce intraoperative risks. In this case, the simulation and preoperative planning have resulted in a good clinical outcome.
